# Intervention effects of a school-based health promotion program on children’s motor skills

**DOI:** 10.1007/s10389-016-0715-x

**Published:** 2016-02-25

**Authors:** Christine Lämmle, Susanne Kobel, Olivia Wartha, Tamara Wirt, Jürgen M. Steinacker

**Affiliations:** Division of Sports and Rehabilitation, Department of Internal Medicine II, Ulm University Medical Centre, Frauensteige 6, Haus 58/33, 89075 Ulm, Germany

**Keywords:** Children, Motor skills, Motor performance, Health promotion, Intervention

## Abstract

**Purpose:**

Physical activity (PA) has positive effects on children’s development. Particularly in childhood, PA plays an important role for children’s motor skills. The objective of this study was to examine the influence of the intervention program “Join the Healthy Boat” on motor abilities of primary school children.

**Methods:**

The baseline measurements of this longitudinal intervention study with an intervention (IG) and control group (CG) were taken at the beginning of the school year 2010/2011 (T1) and follow up measurements in 2011/2012 (T2). Efficacy of the intervention on children’s motor abilities was assessed using a standardized and validated test battery (Dordel-Koch-Test). An exploratory factor analysis was performed in order to reduce dimensions. Differences between CG and IG were examined using analysis of covariance adjusting for age, gender, BMI percentiles and baseline data.

**Results:**

Children in the IG showed an significant improvement in the conditional skills (F(1,1571) = 5.20, *p* ≤ 0.02) and less decline in flexibility (F(1,1715) = 6.68, *p* ≤ 0.01) than children in the CG. Additionally, positive differences in the flexibility tests were ascertained for girls, F(1,839) = 100.88, *p* ≤ 0.02).

**Conclusion:**

The study showed that an intervention that aims to increase PA affects certain parts of children’s motor skills significantly. This was achieved without any extra PA lessons at school but with a low-threshold intervention integrated into the daily school routine.

## Introduction

Worldwide, more than 3.2 million deaths annually are attributable to diseases related to physical inactivity (WHO 2015), which has been linked to increased all-cause mortality (Löllgen et al. 2009) and higher rates of non-communicable diseases (Lee et al. 2012) such as type II diabetes, hypertension, colon cancer, depression and osteoporosis (Pate et al. 1995; Trost et al. 2001; Mammen and Faulkner 2013).

On the other hand, a lot is known about positive effects of regular physical activity (PA). Particularly in childhood, PA is important for children’s healthy growth (Strong et al. 2005; Andersen et al. 2006) and the benefits of PA on motoric, emotional and social development have been established sufficiently (Strong et al. 2005; Andersen et al. 2006). Regular PA promotes, besides perception, concentration and learning abilities children’s motoric performance (Hills et al. 2007; Eime et al. 2013). Especially the formation of fundamental motor skills plays an important role in childhood. Well-pronounced motor abilities are essential for physical fitness (Rivilis et al. 2011) and the foundation for an active lifestyle (Lubans et al. 2010). Several studies report that fundamental motor skill competence is positively related to PA participation in childhood and youth (Haubenstricker and Seefeldt 1986; Wrotniak et al. 2006; Lubans et al. 2010). However, physical inactivity and motoric deficits in childhood are predictors for morbidity in adolescence and adulthood. This can be seen in a special degree of overweight, obesity, muscular-skeletal health problems and cardiovascular diseases (Hollmann and Hettinger 2000; Ketelhut et al. 2005). In the last decades, the decline of PA levels and motor abilities in childhood and youth has made its presence felt (Lämmle et al. 2012; Hallal et al. 2012). Further, since the 1970s, motor abilities have decreased by 10–20 % in almost all aspects (Bös 2003). This is particularly noticeable in German primary school children, a longitudinal database with results of motor ability tests, collected between 1965 and 2006, showed an average decline of nearly 7 % for motor abilities of primary school children in Germany (Bös et al. 2009). Especially total body coordination (Dordel 2000) and endurance performance (Bös 2003) decreased noticeably. To prevent this decline and children’s low PA levels, a preventive strategy is needed which increases children’s motor skills and PA participation. The importance of primary prevention to promote children’s PA for long-term health is well-documented (Myer et al. 2013). Thus, a school-based, teacher-centered health promotion program called “Join the Healthy Boat” was developed. The intervention includes 13 PA teaching units and short daily exercises in class, for 10 to 15 min, to increase PA, which target an increase in children’s motor abilities especially on flexibility, coordinative and conditional skills.

The aim of this paper therefore is to examine the influence of the intervention program “Join the Healthy Boat” on motor abilities of primary school children.

## Methods

### Intervention and evaluation design

The evaluation of this school-based, teacher-centered intervention (“Join the Healthy Boat”) is a prospective, stratified, cluster-randomized, and longitudinal study including an intervention group (IG) and a control group (CG). The study participants were recruited in several ways. For example, information about the study was disseminated by education and health authorities, university schools of education, electronic newsletter, television and radio, and adverts in training catalogs for primary school teachers throughout Baden-Wuerttemberg (South-West Germany).

After the recruiting period, 94 schools were assessed for eligibility. Before randomization three schools declined to participate for unknown reasons; subsequently, 91 primary schools were randomized, 45 schools were allocated to the IG and 46 schools to the CG. One school in the IG withdrew from the participation because of the commitment required for participation was found to be too much, and four schools in the CG because of the randomization in the CG.

At baseline, data of 1,943 first and second grade primary-school children in 157 classes (81 classes in the IG; 76 classes in the CG) were compiled. During the intervention year, one school of the IG withdrew their participation because of unknown reasons and two schools of the CG withdrew due to parental request, while the other school was destroyed by fire. Further information about the recruiting process has been published in Dreyhaupt et al. 2012.

At the beginning of the school year 2010/2011, after baseline measurements were taken, the intervention started in the IG. Simultaneously, the CG followed the regular school curriculum without the intervention. Follow-up measurements (T2) were taken after one intervention year, in the school year 2011/2012. After the T2 measurement, the CG started with the intervention too. Further details about the evaluation design, the program and other aspects of the evaluation study have already been published elsewhere (Kobel et al. 2014; Dreyhaupt et al. 2012). The study was approved by the Ministry of Culture and Education and the university’s ethics committee and is in accordance with the Declaration of Helsinki. The study is registered at the German Clinical Trials Register (DRKS00000494).

### Instruments

Anthropometric measurements such as children’s height (cm) and body weight (kg) were taken by trained staff according to ISAK standards (Stewart et al. 2011) using a stadiometer and calibrated electronic scales (Seca 213 and Seca 826, resp., Seca Weighing and Measuring Systems, Hamburg, Germany). Height was measured to the nearest 0.1 cm and weight was measured to the nearest 0.05 kg, whereas BMI was calculated as weight divided by height squared. Further, to define children’s weight status BMI was converted to BMI percentiles (BMIPCT) using German reference data (Kromeyer-Hauschild et al. 2001). Cut-off points for overweight children were determined above the 90th percentile and for obese children above the 97th percentile.

### Motor abilities test

Children’s motor abilities were assessed with the standardized and validated Dordel-Koch-Test (DKT; Dordel and Koch 2004). The tests were carried out by skilled examiners in small groups. The DKT is a test battery to assess the main types of a child’s motor abilities. It assesses flexibility, conditional and coordinative skills with the following exercises:

1. Conditional skills
  *Standing long jump:* The aim of this test is to determine jumping power. Children had to jump with both legs as far as possible and land on both feet. Each child had two tries; the higher value was used for data analysis (Dordel and Koch 2004).
  *Sit*-*ups*: The purpose of sit-ups is to assess strength and endurance of the abdominal muscles and hip-flexors. Each child had 40 s to do as many sit-ups as possible (Dordel and Koch 2004).
  *Push*-*ups*: Push-ups were used to examine muscular strength and endurance of arms and trunk. Only correctly performed push-ups within 40 s were noted (Dordel and Koch 2004).
  *6*-*min run*: The objective of the 6-min run is to quantify aerobic endurance. The participants ran for 6 min as far as they could, exact distance in meters was recorded (Dordel and Koch 2004).
2. Coordinative skills
  *Lateral jumps:* The aim of this test is to assess whole body co-ordination under time pressure. Each child jumped back and forth over a line as often as possible within 15 s. After two trials, the number of correctly performed jumps of both trials was noted (Dordel and Koch 2004).
  *One-leg stand:* This test is used to examine co-ordination for precision and balance while standing. Children stood barefoot on a small rope on the floor on one leg for 60 s 1 min. The number of times the free leg made ground contact was recorded (Dordel and Koch 2004).
3. Flexibility
  *Sit and reach:* The sit and reach test is a general test of flexibility and specifically assesses the flexibility of the lower back and hamstring muscles. Children’s legs were fully stretched against a standardized sit and reach box when reaching along the top of the box with both hands as far forward as possible. The distance reached by the fingertips (cm) was noted (Dordel and Koch 2004).

### Data analysis

Statistics were performed using SPSS Statistics 21 (SPSS Inc., Chicago, IL, USA) with a significance level set to *p* ≤ 0.05. Descriptive statistics were calculated (mean values and standard deviations). For better interpretation of the motor test battery an exploratory factor analysis (principal components analysis, varimax rotation) was performed in order to reduce dimensions. Therefore, based on the definition of motor abilities of Bös and Mechling (1983), two factors were assumed (coordination and condition). Since flexibility is not defined as a separate motor skill, but an important prerequisite for performance (Bös and Mechling 1983), it was considered separately.

Differences in the three subgroups of motor abilities (flexibility, conditional and coordinative skills) between CG and IG were examined using Analysis of covariance (ANCOVA), adjusting for age, gender, BMIPCT and baseline data; similarly, gender differences in CG and IG were analyzed, also adjusting for age, BMIPCT and baseline.

## Results

At baseline, data of 1,943 first and second grade primary school children (7.1 ± 0.6 years; 51.2 % male) in 157 classes (81 classes in the IG; 76 classes in the CG) were available. Only children who took part in the baseline motor skills testing (T1) as well as follow-up testing (T2) were included in the data analysis; therefore, data of 1,736 participants (7.1 ± 0.6 years; 50.6 % male) were used, constituting 89.2 % of the whole study population.

### Descriptive statistics

Baseline socio-demographic and anthropometric characteristics of the participants are shown in Table 1. Group comparison to check if randomization was successful revealed no differences between CG and IG for any relevant variables. No significant gender differences were found for height, weight, or BMIPCT.

**Table 1:** Baseline characteristics of participants with complete motor ability tests in the “Join the Healthy Boat” study

	Missing values	Intervention (*n* = 957)	Control (*n* = 779)	Total (*n* = 1,736)
Age, years [m (SD)]		7.1 (0.6)	7.1 (0.6)	7.1 (0.6)
Boys, n [%]		471 (49.2)	408 (52.4)	879 (50.6)
First grade, n [%]		486 (50.8)	416 (53.4)	902 (52.0)
Weight, kg [m (SD)]	51	24.9 (5.2)	24.49 (4.6)	24.7 (4.9)
Height, m [m (SD)]	50	124.0 (6.5)	123.71 (6.3)	123.9 (6.4)
BMI, kg/m^2^ [m (SD)]	51	16.0 (2.2)	15.91 (2.0)	16 (2.2)
BMIPCT [m (SD)]	51	49 (27.9)	47.9 (27.4)	48.5 (27.7)
Overweight and obesity,* n* [%]	54	94 (10.2)	66 (8.7)	160 (9.5)

*m S(D)* mean (standard deviation);* BMI* body mass index,* BMIPCT* BMI percentiles

### Factor analysis

The principal components analysis revealed the factors and explained 59.8 % of the variance. The first factor can be interpreted as conditional skills including tests such as standing long jump, sit-ups, push-ups and the 6-min run and the second factor as coordinative skills included the one-leg stand and lateral jump test. The appropriateness of the model is supported by Bartlett’s test (*χ*2 = 2081.62, *p* ≤ 0.000), the Kaiser-Meyer-Olkin criterion (KMO .814) and measure of sampling adequacy (MSA .795 [lowest]).

### Intervention effects on children’s motor performance

For children in the IG, a significantly less decline in flexibility—F(1,1715) = 6.68, *p* ≤ 0.01—and an improvement in the conditional skills, F(1,1571) = 5.20, *p* ≤ 0.02, were ascertained in comparison to the children in the CG. The model explained 54.0 % of variance in flexibility, adjusted* R*^2^ = 0.540, F(5,1715) = 405.00, *p* ≤ 0.000, and 37.6 % of variance—adjusted * R*^2^ = 0.376, F(8,1571) = 120.08, *p* ≤ 0.02—in the conditional skills both adjusted for age, gender, BMIPCT and baseline data. Additionally, girls showed a significant improvement in the flexibility tests—F(1,839) = 100.88, *p* ≤ 0.02. Results are shown in Table 2, while baseline and follow up results for each motoric test are shown in Table 3.

**Table 2:** Intervention effects on children’s motor performance after 1-year intervention: T2-T1 differences

	Intervention group			Control group		
	Boys (*n* = 471)	Girls (*n* = 486)	Total (*n* = 957)	Boys (*n* = 408)	Girls (*n* = 371)	Total (*n* = 779)
Conditional skills^a^						
Standing long jump, cm [m (SD)]	12.0 (19.3)	10.0 (17.5)	11.0 (18.4)	9.2 (18.6)	11.3 (17.5)	10.2 (18.1)
Sit-ups,* n* [m (SD)]	3.3 (6.4)	2.3 (5.9)	2.8 (6.1)	2.5 (6.4)	2.6 (5.5)	2.5 (6)
Push-ups,* n* [m (SD)]	2.9 (5.2)	1.3 (5)	2.1 (5.1)	1.9 (5.1)	1.1 (5)	1.5 (5.1)
6-min run, m [m (SD)]	78.3 (133.6)	62.8 (121.7)	70.5 (127.9)	68.4 (108.1)	48.3 (103.2)	58.9 (106.2)
Coordinative skills						
One-leg stand,* n* [m (SD)]	−2.4 (5.6)	−2.2 (4.5)	−2.3 (5.1)	−2.2 (4.4)	−2 (4.5)	−2.1 (4.5)
Lateral jumps,* n* [m (SD)]	11.3 (9.2)	11.5 (9.6)	11.4 (9.4)	11.1 (8.9)	11.3 (8.9)	11.2 (8.9)
Flexibility^b,c^						
Sit and reach, cm [m (SD)]	−0.5 (4.2)	.3 (4.3)	−0.1 (4.3)	−0.6 (4.6)	−0.2 (4.6)	−0.4 (4.6)

^a^Significant difference between intervention and control group *p* ≤ 0.05

^b^Significant difference between intervention and control group *p* ≤ 0.01

^c^Significant difference between girls in the intervention and control group *p* ≤ 0.02

**Table 3:** Baseline and follow up results for each motoric tests

	Intervention group						Control group					
	Baseline (T1)			Follow-up (T2)			Baseline (T1)			Follow-up (T2)		
	Boys (*n* = 471)	Girls (*n* = 486)	Total (*n* = 957)	Boys (*n* = 471)	Girls (*n* = 486)	Total (*n* = 957)	Boys (*n* = 408)	Girls (*n* = 371)	Total (*n* = 779)	Boys (*n* = 408)	Girls (*n* = 371)	Total (*n* = 779)
Conditional skills												
Standing long jump, cm [m (SD)]	115.6 (±20.2)	109.9 (±20.3)	112.7 (±20.4)	127.5 (±20.6)	119.9 (±19.7)	123.6 (±20.5)	117.4 (±21.6)	109.2 (±21.1)	113.5 (±21.7)	126.5 (±19.8)	120.8 (±19.1)	123.8 (±19.7)
Sit-ups, * n* [m (SD)]	12.3 (±6.2)	11.7 (±5.9)	12.0 (±6.1)	15.7 (6.1)	14.1 (±6.0)	14.8 (±6.1)	12.3 (±6.1)	11.5 (±5.6)	11.9 (±5.9)	15.1 (±6.1)	14.2 (±4.9)	14.7 (±5.6)
Push-ups, * n* [m (SD)]	5.5 (±4.1)	4.9 (±4.0)	5.2 (±4.1)	8.4 (±4.5)	6.1 (±4.1)	7.3 (±4.5)	5.9 (±4.2)	5.1 (±4.0)	5.5 (±4.1)	8.0 (±4.4)	6.2 (±4.0)	7.1 (±4.3)
6-min run, m [m (SD)]^a^	865.31 (±128.8)	813.8 (±110.9)	839.7 (±122.9)	952.8 (±140.8)	882.4 (±125.2)	917.0 (±137.6)	876.5 (±126.7)	831.5 (±107.5)	855.3 (±120.1)	952.1 (±123.6)	879.9 (111.6)	917.7 (±123.4)
Coordinative skills												
One-leg stand,* n* [m (SD)]	5.3 (±6.1)	4.1 (±5.0)	4.7 (±5.6)	2.8 (±3.9)	1.8 (±3.2)	2.3 (±3.6)	5.0 (±5.1)	4.0 (±5.4)	4.5 (±5.3)	2.7 (±3.9)	2.0 (±3.3)	2.3 (±3.6)
Lateral jumps, n [m (SD)]	41.1 (±11.9)	42.6 (±13.1)	41.9 (±12.5)	52.8 (±12.3)	54.3 (12.1)	53.6 (±12.2)	40.7 (±12.7)	40.7 (±12.8)	40.7 (±12.7)	52.1 (±12.9)	52.1 (±11.7)	52.1 (±12.3)
Flexibility												
Sit and reach, cm [m (SD)]^b^	1.2 (±5.7)	2.9 (±5.8)	2.0 (±5.8)	0.6 (±6.1)	3.5 (±6.5)	2.1 (±6.4)	0.5 (±5.5)	2.4 (±5.3)	1.4 (±5.5)	−0.2 (±5.9)	2.3 (±6.0)	1.0 (±6.1)

*m (SD)* mean (standard deviation)

^a^At baseline, the CG and IG differences in the 6-min run are significant (*p* ≤ 0.01); no gender differences were found

^b^At baseline, the CG and IG differences in flexibility are significant (*p* ≤ 0.02); no gender differences were found

## Discussion

This paper investigates the effects of the school-based health promotion program “Join the Healthy Boat” on children’s motor abilities. Whilst the intervention does not only address motor skills, it includes 13 PA teaching units and short daily exercises in class. The mediating effect of the intervention on three relevant motor skills was assessed, namely on coordination, condition and flexibility.

After the 1-year intervention period, significant effects were found with regards to the conditional skills. Children’s conditional skills were assessed using standing long jump, push-ups, sit-ups and a 6-min run, which were also used in other large school-based interventions assessing motor skills in primary school children. Similar results were shown in a 1-year intervention aiming to improve fitness of first graders in Switzerland by adding two extra PE lessons per week (Kriemler et al. 2010). Children in the intervention group showed significant improvements in the 6-min run compared to children in the control group. Similarly, the study from Graf et al. (2005) revealed significant increases in children’s endurance performance after 20 months of a school-based, teacher-centered intervention with additional PA during school mornings; and even more gratifying, comparable results regarding children’s conditional skills could be demonstrated using an intervention without extracurricular physical activity beyond the two short (10—15 min each) exercise breaks performed in class.

Another school-based intervention which increased the amount of PA at school, has shown significant positive effects with regards to coordinative skills (Graf et al. 2005). After 1 year, the 6–9-year-old girls increased their performance in lateral jumping significantly, while for boys, no significant improvement was found. Contrary to the study of Graf et al. (2005), the current analyses revealed no effect on coordinative skills, which might be due to the low-threshold intervention of “Join the Healthy Boat”, which mainly focused on behavioral change on the basis of the opportunity of physical activity. In the current study, coordination was not only assessed by lateral jumps but also in conjunction with a one-leg stand. Balance (as part of coordination) has been shown to be influenced by weight status (Deforche et al. 2009), which was not the case in this study regarding any of the assessed skills.

Furthermore, significant effects were found with regards to flexibility in the whole study population, which was measured by a standardized sit and reach test. Children of the IG showed less decline in their flexibility proficiency than their counterparts in the CG.

Comparing the results with previous research, Sacchetti et al. (2013) also found neither a difference in flexibility after a 2-year school-based intervention in 8–9 year olds, nor after a 3-year intervention in 8–11 year old children (Sacchetti et al. 2015). Similar to the two formerly mentioned studies (Sacchetti et al. 2013, 2015), this program aimed to increase children’s daily PA levels (Kobel et al. 2014), which is known to be associated with children’s motor skills (Laukkaneen et al. 2014).

The intervention showed a significant improvement in flexibility skill for girls; however, for boys, their flexibility could not be influenced positively, only a further decline was reduced compared to the CG. That an increase in girl’s flexibility is possible with an intervention only lasting 6 months has been shown previously (Kain et al. 2004); however, that was with an additional 90 min/week of PA. In contrast to this intervention, the investigated health promotion program “Join the healthy boat” could improve flexibility in girls simply via low intensity exercises.

Nevertheless, the findings of this study should be considered carefully since they may not be generalizable as only South-West Germany was assessed and the sample consists of a rather low overweight prevalence. Although a standardized and validated test battery was used, comparability with other studies which might have used different testing methods may be difficult. Nevertheless, the large sample size, with children from urban and rural schools as well as from diverse backgrounds represents a strength of this study.

Besides, it is imperative to investigate children’s motor abilities and their correlates as well as intervention opportunities, as fundamental motor skills are essential to the development of skills that are further required for sports activities and are positively related to PA participation in childhood and youth (Haubenstricker and Seefeldt 1986; Wrotniak et al. 2006; Lubans et al. 2010). Since early childhood is described as the optimal time to develop motor skills and establish motor competence (Hardy et al. 2010), the school setting is a suitable opportunity for the promotion of children’s PA including their motor abilities.

## Conclusion

The link between motor skills and PA is well documented and during childhood the foundations are laid for an active and healthy lifestyle; therefore, the importance of promoting PA in childhood should not be underestimated.

This study investigated the effects of the school-based and teacher-centered health promotion program “Join the healthy Boat” on the motor abilities of German primary-school children. It was shown that an intervention that aims to increase PA affects certain aspects of children’s motor skills. In particular, an improvement of conditional skills in the IG and advanced flexibility performance in girls became apparent, which was achieved without any extra PA lessons at school but rather with a low-threshold intervention integrated into the daily school routine. In order to achieve improvements in coordinative skills, a longer intervention period may be necessary, which should be investigated in further research.
